# Data on excessive risk of cancer from gamma radiation in residents of Bojnurd city

**DOI:** 10.1016/j.dib.2018.10.052

**Published:** 2018-10-23

**Authors:** Mohsen Khosroabadi, Seyed Abolghasem Haeri, Homa Rezaei Moghaddam, Mohammad Mirdoraghi

**Affiliations:** a Faculty of Medicine, North Khorasan University of Medical Sciences, Bojnurd, Iran; b Assistant Professor, Nuclear Science and Technology Research Institute, Tehran, Iran; c Imam Ali Hospital, North Khorasan University of Medical Sciences, Bojnurd, Iran; d Radiation Protection and Radiobiology Student, Department of Radiology and Radiotherapy, School of Allied Medicine, Tehran University of Medical Sciences, Tehran, Iran

**Keywords:** Gamma, Effective dose, Cancer, Bojnurd

## Abstract

The aim of the data was to measure the absorbed dose of gamma radiation in order to estimate the excessive risk of cancer-induced gamma radiation during the lifetime of Bojnurd residents. In this descriptive cross-sectional study, gamma radiations in 30 places was measured in Bojnurd City during four seasons in 2015. A dosimeter was stacked on a tripod at 1 m from the ground for 50 minutes, and then, the absorbed dose of gamma radiation was recorded in the checklist. Ultimately, the effective dose and the excessive lifetime risk of cancer were determined. The mean ± SE of absorbed dose of gamma radiation in spring, summer, autumn, winter was 134.25 ± 1.45; 139.89 ± 1.64; 134.40 ± 1.25; 143.80 ± 1.73 nGy, respectively. The average annual effective dose by residents in open space was estimated at an average of 0.167 mSv. Also, the excessive risk of cancer-induced gamma radiation was equal to 0.67 × 10^−3^. The annual effective dose and the excessive risk of cancer-induced gamma radiation during the lifetime of Bojnurd residents are higher than the global average.

**Specifications table**

**Table:** 

Subject area	Radiation biology and radiation protection.
More specific subject area	Determine the Excessive Risk of Cancer from Gamma Radiation.
Type of data	Tables.
How data was acquired	To calculate the excessive risk of cancer induced by gamma radiation, a dosimeter was stacked on a tripod at 1 m from the ground for 50 minutes, and then, the absorbed dose of gamma radiation was recorded in the checklist. Ultimately, the effective dose and the excessive lifetime risk of cancer were estimated based on standard equations [1], [2].
Data format	Raw, Analyzed.
Experimental factors	The absorbed dose of gamma and the effective dose were analyzed according to the standards
Experimental features	The absorbed dose of gamma radiation, the effective dose and also the excessive lifetime risk of cancer were determined.
Data source location	Bojnurd, Iran.
Data accessibility	The data are available with this article

**Value of the data**

• The data can be used to show that the amount of the absorbed dose of gamma radiation in residents of Bojnuurd city in open space is more than the global average.
• The data demonstrated that the effective dose from the background radiation was 0.167 Millisievert per year, which is more than two times the universal standard.
• The calculated ELCR is higher than the global average of the risk of cancer-inducing gamma radiation during the lifetime of the residents of Bojnurd.

## 1 Data

The average of all samples in the open environment is 137.10 nGy/h (Nanogray per hour). The maximum of gamma radiation measurement was 255 nGy/h and the lowest value measured was 53 nGy/h [Table 1]. The ANOVA and Scheffe post hoc test were shown that there is a significant relationship between measured gamma radiation in winter with the measured values in autumn and spring (*P* < 0.05). There was no any significant relation between other seasons (*P* > 0.05) [Table 2].

**Table 1:** The mean, Std. Error, maximum, minimum of measured gamma radiation (nGy), the effective dose (mSv) and ELCR per year are shown with 95% Confidence Interval for Mean.

**Season**	**N**	**Mean**	**Std. Error**	**95% Confidence interval for mean**		**Minimum**	**Maximum**	**The effective dose (mSv)**	**ELCR** × **10^-3^**
				**Lower bound**	**Upper bound**				
Spring	360	134.25	1.45619	131.3835	137.1110	65.00	255.00	0.0411	0.164
Summer	360	139.89	1.64803	136.6451	143.1271	53.00	233.00	0.0428	0.171
Autumn	360	134.40	1.25166	131.9357	136.8587	77.00	206.00	0.0412	0.164
Winter	148	143.80	1.73549	140.3743	147.2338	89.00	215.00	0.0440	0.175
Total (Annual)	1228	137.10	.77632	135.5730	138.6191	53.00	255.00	0.168	0.67

**Table 2:** Comparison between measured gamma radiations in four seasons by Scheffe post hoc test.

**(I) Season**	**(J) Season**	**Mean difference (I-J)**	**Std. Error**	**Sig.**	**95% Confidence Interval**	
					**Lower bound**	**Upper bound**
Spring	Summer	−5.63889*	2.01333	.050	−11.2750	−.0028
	Autumn	−.15000	2.01333	1.000	−5.7861	5.4861
	Winter	−9.55683*	2.63755	.004	−16.9403	−2.1733
Summer	Spring	5.63889*	2.01333	.050	.0028	11.2750
	Autumn	5.48889	2.01333	.060	−.1472	11.1250
	Winter	−3.91794	2.63755	.531	−11.3015	3.4656
Autumn	Spring	.15000	2.01333	1.000	−5.4861	5.7861
	Summer	−5.48889	2.01333	.060	−11.1250	.1472
	Winter	−9.40683*	2.63755	.005	−16.7903	−2.0233
Winter	Spring	9.55683*	2.63755	.004	2.1733	16.9403
	Summer	3.91794	2.63755	.531	−3.4656	11.3015
	Autumn	9.40683*	2.63755	.005	2.0233	16.7903

* The mean difference is significant at the 0.05 level.

According to the Eq. (3), the annual effective dose for residents of Bojnurd city was equal to 0.168 mSv. Based on the following equation, the risk of inducing cancer by receiving gamma radiation can be calculated [4], [5].

(1) $$ELCR=AED\times D_{L}\times R_{F}$$

ELCR = Excess Lifetime Cancer Risk
*E* = Annual effective dose in mSv
*D*_L_ = Average lifespan (year)
*R*_F_ = Fatal risk factor per Sievert for the public (0/057 Sv^−1^).
ELCR = 0.168 ×70/1 × 0/057 = 0.67 × 10^−3^

## 2 Experimental design, materials and methods

**Fig. 1:**
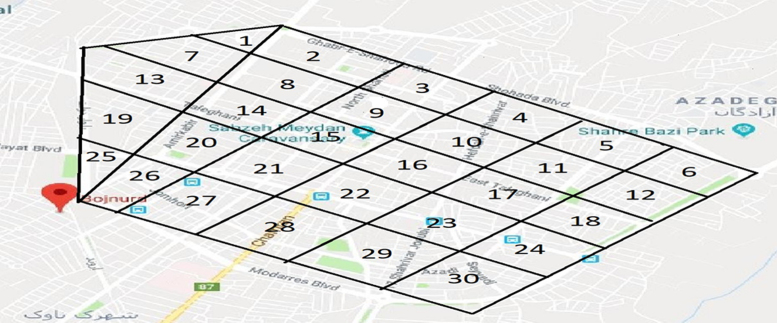
The 30 places in Bojnurd, where the absorbed dose of gamma radiation was measured [3].

The data were derived from a descriptive cross-sectional study, which was conducted in Bojnurd. To select the measuring sites, a comprehensive map of Bojnurd City was prepared according to the distribution of population [Fig. 1]. 30 places were considered. A dosimeter was installed at one meter from the ground along the main directions of northern, southern,eastern, western and sub-directions on a tripod in an open environment to examine the gamma background radiation. Selected locations for measurement were flat, no trees, buildings or walls in that area.

The dosimeter was carried out by the Inspector Survey Meter, which can monitor gamma, beta, x-ray and alpha radiations. The operating range is 0.1 to 1000 µSv/hr. Data were recorded in the checklist and then analyzed by SPSS 16. In order to determine the significant differences between seasons, the data were analyzed by ANOVA and Scheffe post hoc test with statistical confidence interval of 95%. For the purpose of evaluating the effect of ionizing radiation on human biological systems, the International Commission on Radiological Protection (ICRP) is considered equivalent dose which defined based on absorbed dose. Using the following equation, the annual effective dose of gamma radiation of Bojnurd City, which is the sum of cosmic radiations and radiations emitted from radioactive substances in the earth׳s crust, was calculated [8], [9].

(2) $$E\left(Sv\right)=C\left(0.2\;D_{out}\right)\times T$$

In this case, E is an effective dose, C is the conversion factor of absorbed dose to effective dose which is 0.7, T is the conversion factor of hour to year, D_out_ is the absorbed dose of gamma radiations in open space. 0.2 is also related to open space occupancy factor [4], [5], [6], [7]. Ultimately, based on the following equation, the risk of inducing cancer by receiving gamma radiation was calculated [10], [11].

(3) $$ELCR=AED\times D_{L}\times R_{F}$$

ELCR = Excess Lifetime Cancer Risk
*E* = Annual effective dose in mSv
*D*_L_ = Average lifespan (year)
*R*_F_ = Fatal risk factor per Sievert for the public (0/057 Sv^−1^).
