# Supplementary material for: Data on excessive risk of cancer from gamma radiation in residents of Bojnurd city
Source: Data Brief. 2018 Oct 23;21:790–4. doi: 10.1016/j.dib.2018.10.052 (PMC6216078; doi:10.1016/j.dib.2018.10.052)
Supplement: Supplementary file 1 — Supplementary material [file mmc1.docx]

To the editor, 25^th^ Sep 2018

**Data in Brief**

Dear editor,

There is no any conflict of interest for the manuscript entitled, “**Data on Assessment of Excessive Risk of Cancer Induced by Gamma Radiation in Residents of Bojnurd City*”*** All authors declare that.

Thank you in advance for receiving our manuscript to review and your valuable time. Looking forward to hear from you soon.

Sincerely Yours,
*Mohammad Mirdoraghi*
